# Comprehensive Comparisons of Satellite Data, Signals, and Measurements between the BeiDou Navigation Satellite System and the Global Positioning System ^†^

**DOI:** 10.3390/s16050689

**Published:** 2016-05-13

**Authors:** Shau-Shiun Jan, An-Lin Tao

**Affiliations:** Department of Aeronautics and Astronautics, National Cheng Kung University, Tainan 70101, Taiwan; taoanlin@gmail.com

**Keywords:** Global Navigation Satellite System (GNSS), BeiDou Navigation Satellite System (BDS), Global Positioning System (GPS), navigation data, ephemeris, almanac, signal, measurement

## Abstract

The Chinese BeiDou navigation satellite system (BDS) aims to provide global positioning service by 2020. The combined use of BDS and Global Positioning System (GPS) is proposed to provide navigation service with more stringent requirements. Actual satellite data, signals and measurements were collected for more than one month to analyze the positioning service qualities from both BDS and GPS. In addition to the conversions of coordinate and timing system, five data quality analysis (DQA) methods, three signal quality analysis (SQA) methods, and four measurement quality analysis (MQA) methods are proposed in this paper to improve the integrated positioning performance of BDS and GPS. As shown in the experiment results, issues related to BDS and GPS are resolved by the above proposed quality analysis methods. Thus, the anomalies in satellite data, signals and measurements can be detected by following the suggested resolutions to enhance the positioning performance of the combined use of BDS and GPS in the Asia Pacific region.

## 1. Introduction

An increasing number of countries have focused on the development of their own Global Navigation Satellite System (GNSS), which provides convenient real-time positioning, velocity, and time services [1]. Currently, the Global Positioning System (GPS) operated by the United States and Global Navigation Satellite System (GLONASS) operated by Russia provide global positioning services to users [2,3]. In addition, the European Galileo and Chinese BeiDou Navigation Satellite System (BDS), both under development, aim to provide global positioning service by 2020. The Japanese Quasi-Zenith Satellite System (QZSS) and the Indian Regional Navigation Satellite System (IRNSS) are regional navigation satellite systems (RNSS). Different GNSSs have been combined to provide users with more complete and diverse satellite navigation services [4,5].

**Figure 1:**
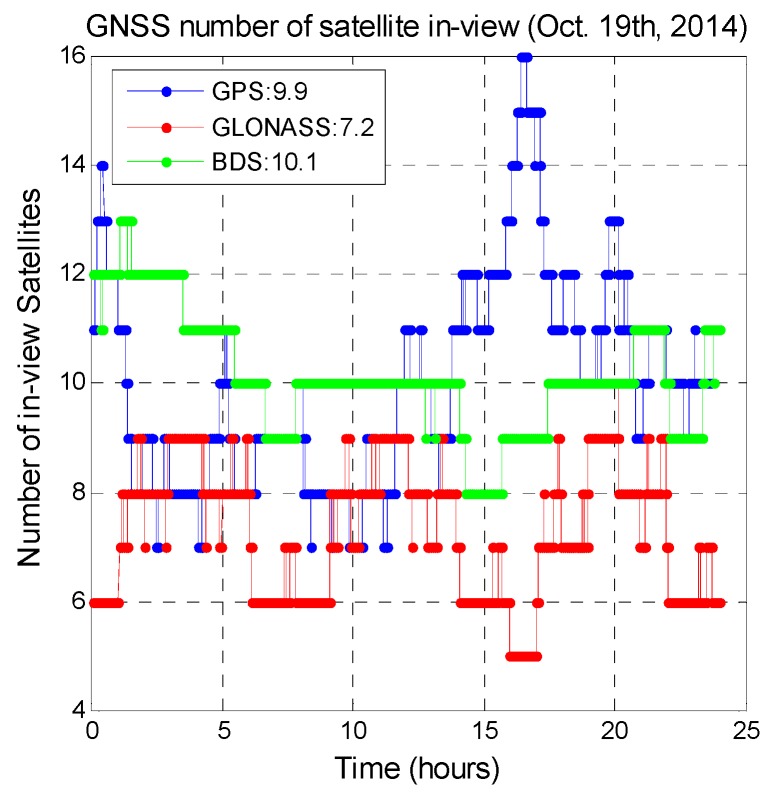
Number of available GPS, GLONASS, and BDS satellites for users in Taiwan.

**Figure 2:**
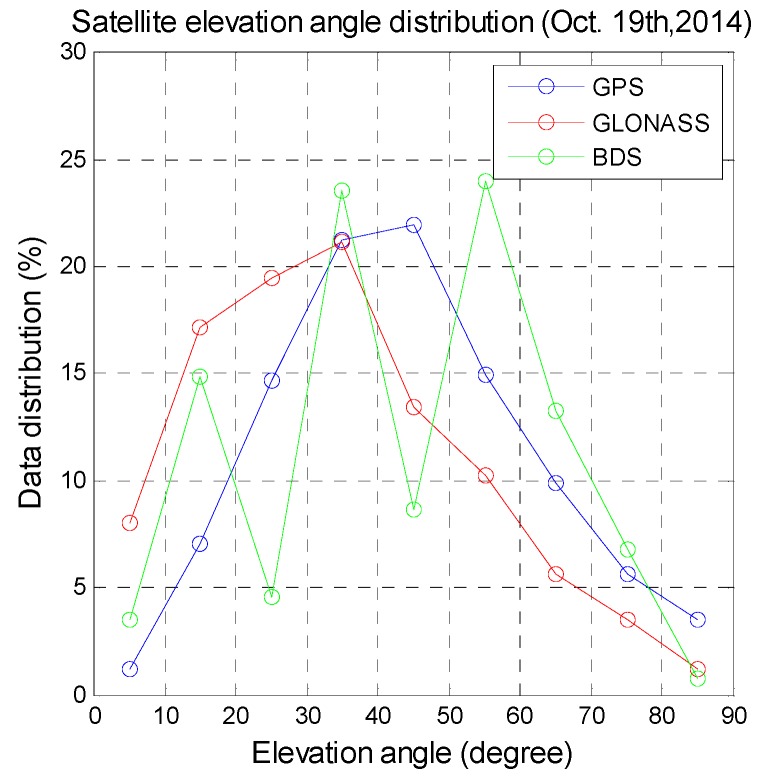
GPS, GLONASS, and BDS satellite distributions for various elevation angles for users in Taiwan.

A regional GNSS is designed to provide unique benefits for a certain area. For users in the Asia Pacific region, GPS, GLONASS, and BDS provide stand-alone positioning services at all times. Galileo is being built up and can thus currently provide only restricted positioning services in this region. For users in urban areas, the integration of different GNSS offers higher positioning availability. Figure 1 shows in-view satellite numbers for various GNSSs generated from the signal in space (SIS) data collected by a user in Taiwan. The data length is one day and the data were collected on the 19th of October 2014. BDS has the highest average number of available satellites. Figure 2 shows the three GNSS satellite distributions at various elevation angles in increments of 10° generated from the data used for Figure 1. More than 50% of the BDS satellites have an elevation angle above 50°. For GLONASS, more than 50% of satellites have elevation angles below 30°. Satellite signal and measurement quality are positively correlated with the satellite elevation angle, and thus BDS provides better measurements for positioning for users in this region.

Integration of several GNSS constellations could enhance the navigation services with better accuracy, continuity and availability than that of one GNSS constellation [6,7]. Especially for environments where some portions of the sky are blocked, for instance, urban canyons and dense foliage environments, the user position cannot be calculated due to the insufficient number of satellite in view. On the other hand, the combined use of GNSSs could provide sufficient signals and therefore provide uninterrupted positioning service in the same environment. Moreover, more visible satellites from multiple GNSSs offer better satellite geometry for the positioning than single GNSS [8]. However, the different GNSS architectures may cause problems during system integration [9,10], so before integrating BDS with GPS, the differences between these systems must be studied.

During the experimental period presented in this paper (10th of July to 13th of August 2014), the BDS service area is 55°S~55°N, 70°E~150°E [11]. The BDS constellation includes five geostationary Earth orbit (GEO) satellites, five inclined geosynchronous orbit (IGSO) satellites, and four medium Earth orbit (MEO) satellites. The different BDS satellite types (*i.e.*, GEO, IGSO, and MEO) are operated in different corresponding orbit altitudes. On the other hand, GPS has 31 MEO satellites and all GPS satellites are operated in similar orbit altitudes. The orbit altitude of GPS satellites is approximately 20,200 km and that of BDS MEO satellites is 21,528 km. The period of GPS satellites is 1/2 sidereal day, approximately equal to 11 h and 58 min. After two periods (23 h and 56 min), the GPS satellites appear at almost the same place. Since the BDS MEO satellites are at a higher orbit altitude than that of GPS satellites, the period of BDS MEO satellites is 7/13 sidereal day, approximately equal to 12 h and 55 min [11]. As a result, the BDS satellite geometry repeats every seven sidereal days for a fixed user.

The system differences between BDS and GPS are summarized in Table 1. This research uses the GPS time (GPST) as the timing system to evaluate the results of BDS, GPS, and their integration (*i.e.*, BDT = GPST − 14) [11]. The coordinate systems for these two systems are based on the Earth-centered Earth-fixed (ECEF) coordinates, and China Geodetic Coordinate System 2000 (CGCS 2000) is used for BDS and GPS uses the World Geodetic System 1984 (WGS84). According to Cheng [12]) the difference between the two coordinate systems is caused by that their definitions of the geocentric gravitational constant (*μ*) and the rate of Earth rotation (*Ω_e_*) are different, and the maximum latitude and longitude differences for these two coordinates are less than 1.1 × 10^−3^ m, so the coordinate conversion effect is thus ignored in this paper.

Under ideal conditions, the coordinate and timing conversions between different GNSSs could be calculated, and the simulation results of [12] shows that integration of two or more GNSSs offers better positioning performance than a single GNSS. However, there are many issues that need to be resolved if one would like to integrate actual signals from two or more GNSS constellations. For most of the multi-GNSS user positioning algorithms, the minimum requirement is to conduct an inter-system bias calibration [13,14]. For example, when a user attempts to combine the measurements from several GNSS constellations to achieve precise positioning, the inter-system biases between different GNSSs would affect the resolution of integer ambiguity [15]. Because the BDS constellation includes MEO, IGSO, and GEO satellites, a BDS receiver has to consider the inter-satellite-type biases between different constellations [16]. Besides these inter-system and inter-satellite-type biases, it is of practical interest to evaluate the differences between BDS and GPS based on actual satellite data, signals and measurements. Moreover, suggestions are provided for combining the two systems for positioning.

This research mainly focuses on finding all the system differences that cause bad positioning results when using combined GPS and BDS data. By analyzing real data, signals, and measurements, the actual performance of a given GNSS can be revealed. Comparisons of GPS and BDS on satellite data, signal and measurements give us a good understanding about their differences. For a complete survey of the performance of BDS and GPS, this research divides the analysis methods into data quality analysis (DQA), signal quality analysis (SQA), and measurement quality analysis (MQA). These analyses correspond to satellite broadcasts of satellite location information, signal arrival at the receiver, and receiver calculation of ranging measurements based on the received signal and receiver’s ability, respectively.

The satellite data, signals, and measurements used in this research were recorded from the 10th of July to the 13th of August 2014. The GPS and BDS signals were recorded at the same time on the roof of the building of the Department of Aeronautics and Astronautics, National Cheng Kung University, Taiwan. The GNSS antenna was a NovAtel GPS-703-GGG, and the receiver was a NovAtel FlexPak6. In order to present the original performance of GPS and BDS, this research used the raw measurement from the NovAtel receiver without any smoothing filter. The raw data was tested in Matlab R2014a on a 3.4-GHz Intel^®^ Core (TM) i7-3770 CPU with 4 GB of RAM. Section 2, Section 3 and Section 4 detail DQA, SQA, and MQA, respectively. GPS and BDS SIS data were analyzed using these three analysis methods.

## 2. Data Quality Analysis

**Figure 3:**
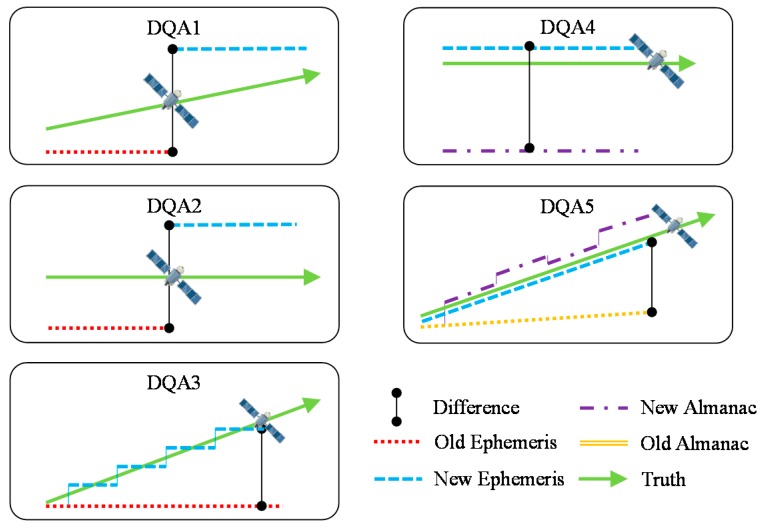
Schematic diagram of five DQAs.

This research firstly determines whether the received satellite navigation data are reliable. GPS and BDS use the Kepler orbit element for calculating satellite position, and thus there will be orbit and clock errors due to the fact the elements in the navigation data not presenting the true activity of satellites [1,17]. By validating the ephemeris and almanac data for each satellite, the difference in satellite control ability and consistency between GPS and BDS was determined. GPS is regarded as a standard for comparing SIS data quality with BDS [18]. Five DQA methods were proposed in our previous research [19], as shown in Figure 3.

They are:

1. DQA1: Satellite position difference when ephemeris is updated.
2. DQA2: Satellite clock correction difference when ephemeris is updated.
3. DQA3: Ephemeris applicable period.
4. DQA4: Satellite position difference between almanac and ephemeris.
5. DQA5: Almanac applicable period.

The navigation data used in DQA analysis were recorded from the 10th of July to the 13th of August 2014. This research uses the raw broadcast navigation data from the receiver without any filtering or data transformation. In the following analysis, the statistical results were estimated using all the data.

### 2.1. DQA1: Satellite Position Difference When Ephemeris Is Updated

DQA1 uses the ephemeris data to estimate the satellite position difference when the ephemeris is updated. When the ephemeris is updated, this analysis uses the original and new ephemeris to calculate the satellite positions. Ideally, the difference in satellite position before and after the ephemeris update should be small if the satellite control ability of the GNSS is stable. The results of DQA1 are shown in Table 2. In DQA1, both systems have the same level of stability most of the time. However, one of BDS IGSO satellites (PRN 10) and two of BDS MEO satellites (PRN 11 and PRN 12) exhibit dramatic differences for some ephemeris updates, and both the new and original ephemerides are declared as healthy. This sudden change in satellite position would degrade user positioning performance. Based on the DQA1 results, the orbit prediction for BDS is not as stable as that of GPS for the experiment period. For a standard GNSS receiver, it is difficult to identify which of the two inconsistent orbits is more accurate. As a result, the DQA1 method is proposed to be included in the BDS-GPS receiver design to validate each satellite position used for positioning and detect the irregular or large change in satellite position because of ephemeris update. If the DQA1 detects any irregularities when ephemeris updates, the corresponding satellites would be excluded from positioning.

### 2.2. DQA2: Satellite Clock Correction Difference When Ephemeris Is Updated

The satellite clock correction for a given satellite is broadcasted in the ephemeris, and the difference of the satellite clock correction between the ephemeris update should be small if the GNSS satellite control ability is stable. It is necessary to analyze the satellite clock correction difference between each ephemeris update, and the DQA2 method is therefore proposed in this work. The results for DQA2 are summarized in Table 3. In this analysis, GPS has stable performance for controlling the satellite clock correction, and all GPS satellites clock correction differences are less than 0.35 m. On the other hand, the differences of the BDS satellite clock correction between ephemeris updates are larger than those of GPS. The clock corrections for BDS satellites need to be improved. As a result, a BDS-GPS user could follow the satellite clock correction estimation method provided in [20] to reduce satellite clock error effect on positioning.

### 2.3. DQA3: Ephemeris Applicable Period

According to [2,11], the GPS satellite updates its ephemeris message every two hours and the BDS satellite updates it every hour. If a receiver does not receive the regular ephemeris update, then the original received ephemeris should be removed after a certain time, because the original received ephemeris is invalid to estimate satellite location and clock. In DQA3, this research selects one specific ephemeris message and uses that for six hours to analyze the satellite location in comparison with that of the updated ephemeris. As shown in [19], most of the GPS satellite position errors increase quickly after using the same ephemeris for more than four hours, and on the other hand, most of the BDS satellite position errors rise rapidly after applying the same ephemeris for three hours. Therefore, the applicable period of ephemeris is four hours for GPS and three hours for BDS.

### 2.4. DQA4: Satellite Position Difference between Almanac and Ephemeris

Almanac data is not as precise as ephemeris data for the satellite position calculation, but the applicable period of almanac data can cover several months. Almanac data is used to accelerate the time-to-first-fix (TTFF) when one turns on a GNSS receiver. In DQA4, we compare the satellite position calculated from the almanac data with the satellite position calculated from the corresponding ephemeris data, and the distribution of the differences of the calculated satellite positions are listed in Table 4. As shown in Table 4, the satellite positioning performance of BDS almanac data is similar to that of GPS almanac data.

### 2.5. DQA5: Almanac Applicable Period

Almanac data could be applied for an extended period of time, but they do have applicable period limitations. The DQA5 use a method similar to DQA3 to find the applicable period of almanac data for both systems, and the test data used in DQA5 is over a month. The almanac is to provide rough satellite locations and the satellite Doppler shift information. The acquisition process can be expedited if the initial Doppler shift guess is close to the true value. The Doppler shift is the relative movement between the satellite and receiver. For a fixed GNSS user, the velocity could be assumed to be due to the satellites only. The maximum Doppler shift for a conventional GNSS receiver could be set to be the maximum radial velocity of the satellites. With the assumptions made in our previous research [19], the maximum radial velocity of GPS was about 1427 m/s, and that of BDS was about 1440 m/s. Finally, DQA5 uses the satellite velocity, position, and elevation angle errors to determine the applicable period of almanac data. Table 5 shows the statistical results for the same almanac used for over five weeks. The velocity errors of both systems at the fifth week are significantly larger than the threshold. Additionally, the BDS IGSO satellites have the largest velocity errors than the other satellites, and the BDS IGSO satellite positioning errors are significantly larger than the other satellites. In addition to the satellite velocity and position errors mentioned above, the satellite elevation error has a more serious impact on the GNSS receiver. The BDS IGSO satellite elevation angle error at the fifth week was over 2°, and this 2° error might influence a GNSS receiver to search proper satellite in view. As shown in Table 5, the GPS almanac data could be applied for more than five weeks. In contrast, the almanac data for BDS IGSO satellite need to be updated more frequently to provide correct information.

### 2.6. DQA Conclusions

According to the results of DQA methods, the qualities of BDS ephemeris and almanac data are similar to that of GPS most of the time. However, sometimes BDS has some irregular ephemeris and almanac information. A BDS/GPS receiver is thus suggested to include the above DOA methods to obtain the integrated positioning with sufficient quality.

## 3. Signal Quality Analysis

Signals are continuously broadcast by satellites. The broadcast signal strengths are the same for all satellites. However, the user receives discontinuous and varying-strength signals from satellites due to free-space path loss and interference in the transmission path. For a given user, each GNSS satellite faces a different environment when transmitting a signal to the receiver. The transmission distance and the interference in this path vary with satellite. When the received signal power is significantly below specified levels, ranging error increases because the autocorrelation function causes error in the code tracking loop [21,22,23,24]. Carrier-phase measurements are more precise than pseudorange measurements, but integer ambiguity is a problem. If the integer ambiguity can be resolved, centimeter-level positioning can be achieved [25]. However, sometimes a GNSS receiver cannot continue to generate carrier-phase measurements due to the temporarily loss of lock on the carrier of a GPS signal caused by signal blockage. A satellite signal may be interrupted by radio interference, irregular ionospheric activity, or objects such as buildings or trees. High receiver dynamics can also cause signal lock loss. When receiver lock on satellite signals is lost, the receiver needs to recalculate the integer ambiguity for the satellite. If the integer ambiguity changes for a satellite, there will be a discontinuity or jump in the carrier-phase measurement. A cycle slip is an indicator of GNSS receiver loss of satellite signals. Receivers need to assign extra time for calculation of the new ambiguity value. As a result, availability is decreased. Analyzing the signal continuity for each satellite can provide useful information for precise positioning. This research analyzes the sensitivity of the received BDS and GPS signals for a given user. The signal strength and continuity differences between BDS and GPS are given. The carrier-to-noise density ratio (C/N_0_) is used to show the signal strength and quality. The C/N_0_ output by a receiver is used as an indicator of signal strength and quality of the tracked satellite and the noise density as seen by the receiver’s front-end [26]. The cycle slip number of the carrier-phase measurements for each satellite is used to analyze the continuity for these two systems. SQA is divided into three parts:

1. SQA1: Signal strength for various times and satellite elevation angles.
2. SQA2: Signal continuity analysis.
3. SQA3: Signal discontinuity analysis.

The data used in this section were recorded on the 22nd of July 2014 for 24 h. The raw data from the NovAtel receiver were recorded at a rate of 1 Hz. The total numbers of data points for GPS and BDS were 870,874 and 899,137, respectively.

### 3.1. SQA1: Signal Strength for Various Times and Satellite Elevation Angles

**Figure 4:**
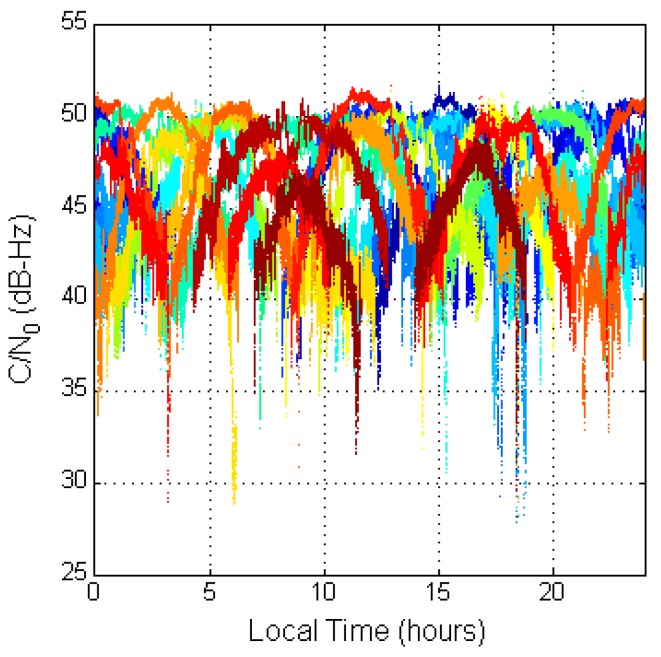
GPS C/N_0_ with respect to local time.

SQA1 is used to confirm the performance of the general received signal strength and find the differences between the two systems. Figure 4 shows the GPS C/N_0_ result for all GPS satellites during a day. The different colors stand for the different GPS satellites. The results are presented to local time in Taiwan (UTC + 8). The result for each satellite is shaped like a parabola due to signal strength becoming stronger when the elevation angle is high and weaker when the elevation angle is low. For GPS satellites, the maximum C/N_0_ is above 50 dB-Hz. This good-quality signal can be received all day.

**Figure 5:**
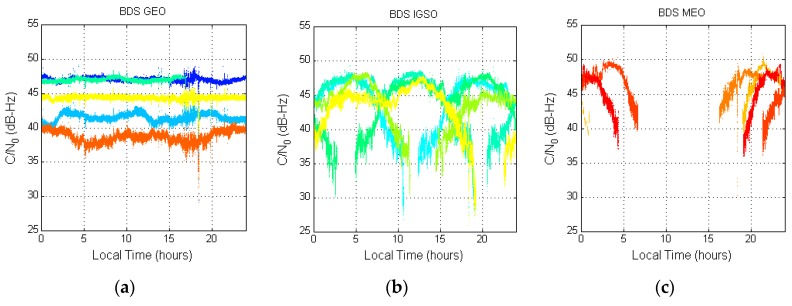
BDS C/N_0_ with respect to local time. (**a**) BDS GEO C/N_0_; (**b**) BDS IGSO C/N_0_; (**c**) BDS MEO C/N_0_.

Figure 5 shows the BDS C/N_0_ results. The BDS satellites have a longer transmission range than that of the GPS satellites, so their signal strength is weaker. The location of the BDS GEO satellites does not change much. As a result, the signal strength of each GEO satellite is rather constant at a specific value. However, this value and the amplitude are different for different GEO satellites due to differences in their elevation angle. The IGSO C/N_0_ shape is not a parabola but it also changes with the satellite elevation angle. The MEO satellites have the shortest distance. Their C/N_0_ value is greater than those of the IGSO and GEO satellites. The maximum C/N_0_ for the whole BDS constellation is below 50 dB-Hz. When there are no MEO satellites, the highest C/N_0_ for non-MEO BDS is only 47 dB-Hz.

**Figure 6:**
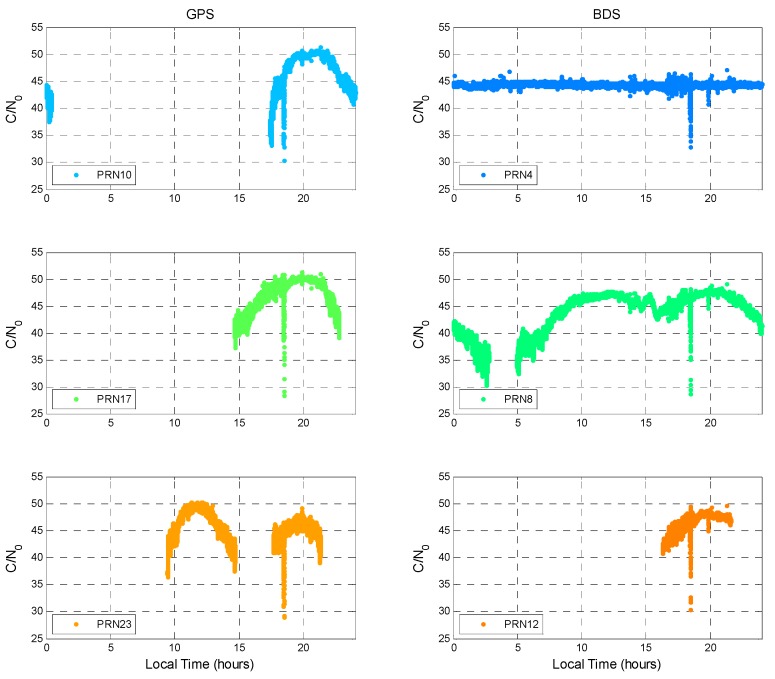
Impact of interference on GPS and BDS.

Our previous work found that the signal received on the 22nd of July 2014 was affected by local interference and that the signal power had an anomalous value [27]. The user receives the interference signal at a local time of around 18:30. All the GPS satellite C/N_0_ values dropped at the same time. Figure 6 shows three GPS satellites at different elevation angles.

C/N_0_ drops when the interference signal is received. On the right-hand side of Figure 6, one GEO satellite, one IGSO satellite, and one MEO satellite are used to demonstrate how the interference signal affects the BDS signal. C/N_0_ decreases for all BDS satellites at the same time. As a result, the interference signal also influences the BDS signal as the center frequency of BDS B1I is close to that of GPS L1.

**Figure 7:**
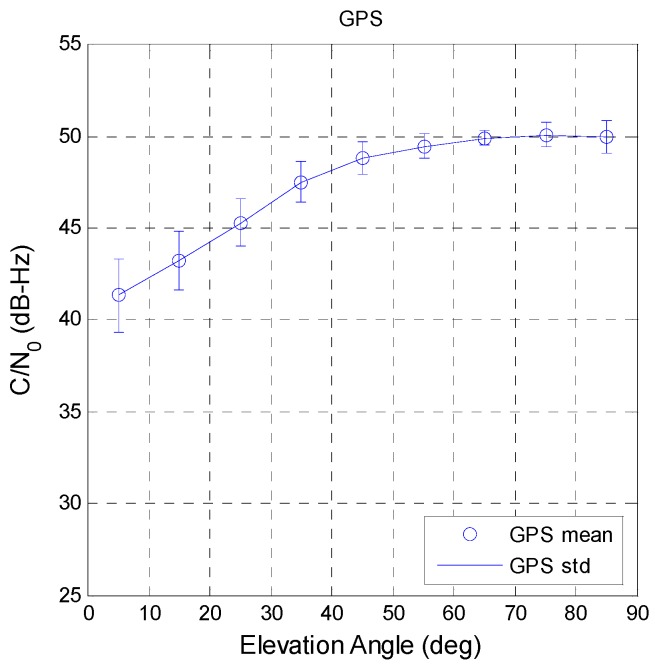
GPS elevation angle with respect to C/N_0_.

The C/N_0_ values for various satellite elevation angles were analyzed. Low-elevation-angle signals pass through a more complicated environment, and thus have more noise. As a result, the C/N_0_ value increases with increasing elevation angle of the satellite. Different GNSS antennas have different gain patterns, and in order to compare the C/N_0_ variations for GPS and BDS, the same GNSS antenna is used for GPS and BDS in the paper. Figure 7 shows the GPS satellite C/N_0_ change with the satellite elevation angle. When the elevation angle was above 60°, the C/N_0_ value remains at its highest value. The blue circles and lines are the mean and standard deviation values for 10° bins. Figure 7 also shows that the distribution of C/N_0_ values becomes wider with decreasing satellite elevation angle.

**Figure 8:**
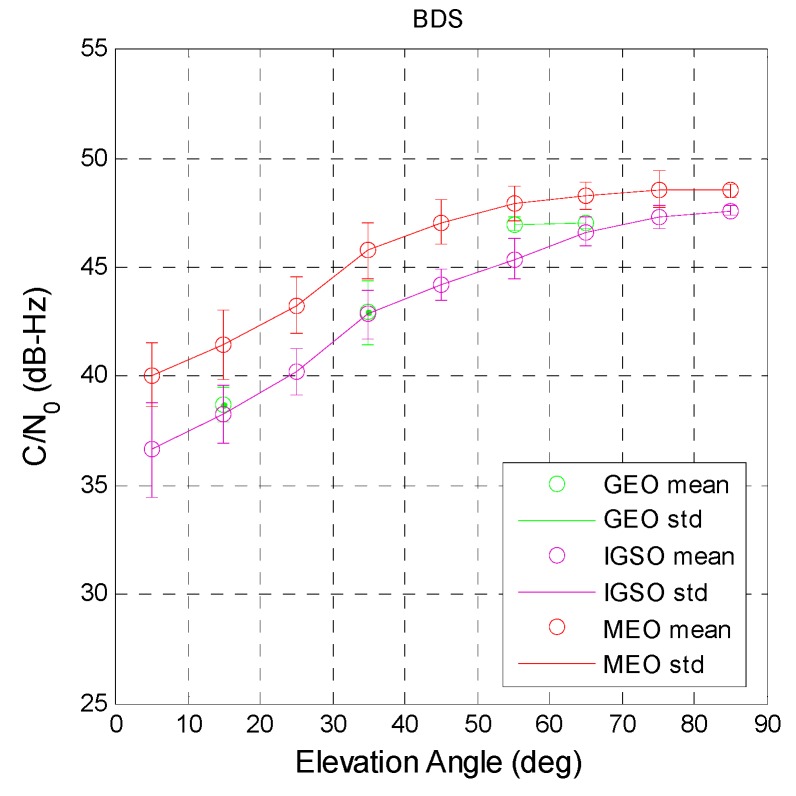
BDS GEO elevation angle with respect to C/N_0_.

Figure 8 shows the three BDS constellation C/N_0_ as a function of elevation. For GEO satellites (green), the results could not be obtained for all elevation angles due to them being almost fixed at a certain location. For users in Taiwan, the approximate elevation angles of GEO satellites PRN 1 to 5 are 55°, 38°, 60°, 38°, and 18°, respectively. The BDS PRN 1 and 3 have the highest satellite elevation angles. Although the C/N_0_ values are lower than those for GPS, the standard deviation is smaller. This indicates that the signal of BDS GEO satellites is more stable than that of GPS. The purple circles and lines in Figure 8 are the C/N_0_ results of BDS IGSO satellites. The highest satellite elevation angle is around 80°. The signal strength of IGSO satellites is weaker than that of GPS satellites at every elevation angle bin. However, the standard deviation shows that the C/N_0_ stability of BDS IGSO satellites is better than that of GPS satellites. Finally, the red circles and lines are the BDS MEO C/N_0_ values, which change with the satellite elevation angle. The signal strength of BDS MEO satellites is better than that of GEO and IGSO satellites but weaker than that of GPS satellites. However, the standard deviation of the C/N_0_ values at each elevation angle bin is the worst of all satellite constellations. The statistical results for the two systems are summarized in Table 6. The table provides the standard C/N_0_ for the user to confirm that received satellite signal power is within normal condition. If the signal power is significantly lower than the statistic result and the user ignores this result, ranging errors may increase and present a greater position error for the user.

### 3.2. SQA2: Signal Continuity Analysis

The discontinuity in the carrier tracking loop generates different integer ambiguity values and causes reduction in user navigation continuity. Cycle slips can occur for one or more epochs. Thus, all cycle slips during the experiment period are counted for each satellite. This section uses the number of measurement epochs without cycle slip compared to the total received data as a continuity indicator for each satellite.

**Figure 9:**
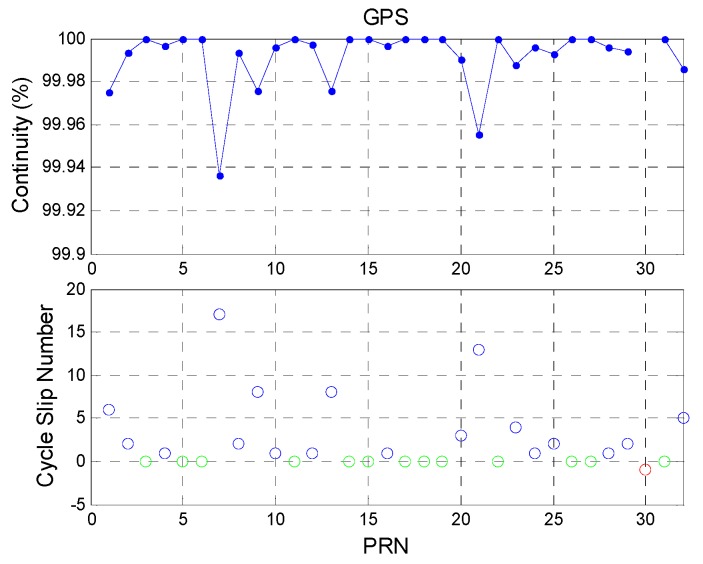
GPS continuity and cycle slip number.

Figure 9 shows the continuity analysis for GPS satellites. The upper figure shows the continuity value for each GPS satellite. The mean value of GPS satellite continuity is 99.9914%. The lower figure shows the total number of cycle slips for each GPS satellite during the experiment period. The green circles indicate that the satellite had no cycle slips during the day (and thus 100% continuity). The red circle indicates that receiver did not receive data from PRN 30. GPS PRN 6 had the most cycle slips and the cycle slip number is 17 epochs during a day.

**Figure 10:**
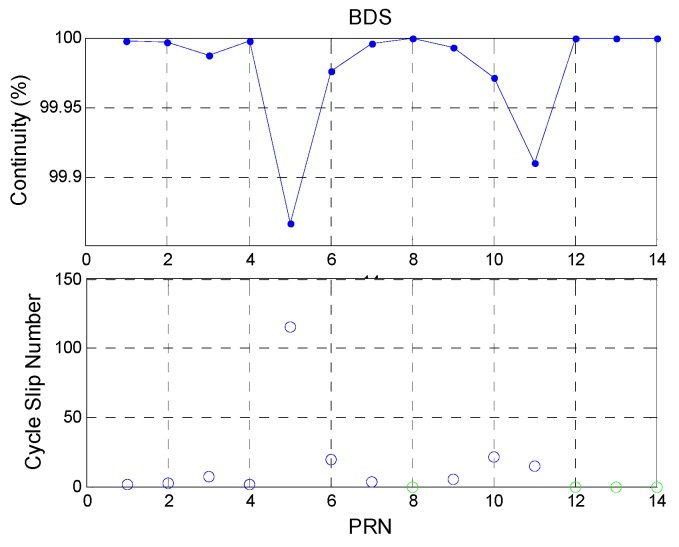
BDS continuity and cycle slip number.

Figure 10 shows the continuity analysis for BDS satellites. The total BDS constellation continuity is 99.9785%, which is lower than that for GPS. This is due to several reasons. First, the BDS GEO PRN 5 satellite had the lowest elevation angle and lost lock all the time. The other GEO satellites had better continuity. Thus, the statistical results for BDS GEO PRN 5 are given separately in order to clarify its impact. Second, the BDS IGSO PRN 6 and 10 satellites had poorer continuity than that of the other IGSO satellites. In addition to the BDS GEO PRN 5, the cycle slip number of BDS IGSO is the highest of all BDS satellites. Finally, the BDS MEO PRN 11 satellite lost lock for a while. The remaining BDS MEO satellites had 100% continuity. As a result, the BDS GEO and IGSO satellites had poorer continuity than the BDS MEO satellites. Because the user position is static and the GPS and BDS signals are received in the same environment at the same time, the discontinuity result of BDS satellites may have been caused by the BDS satellites. The statistical results are shown in Table 7.

### 3.3. SQA3: Signal Discontinuity Analysis

**Figure 11:**
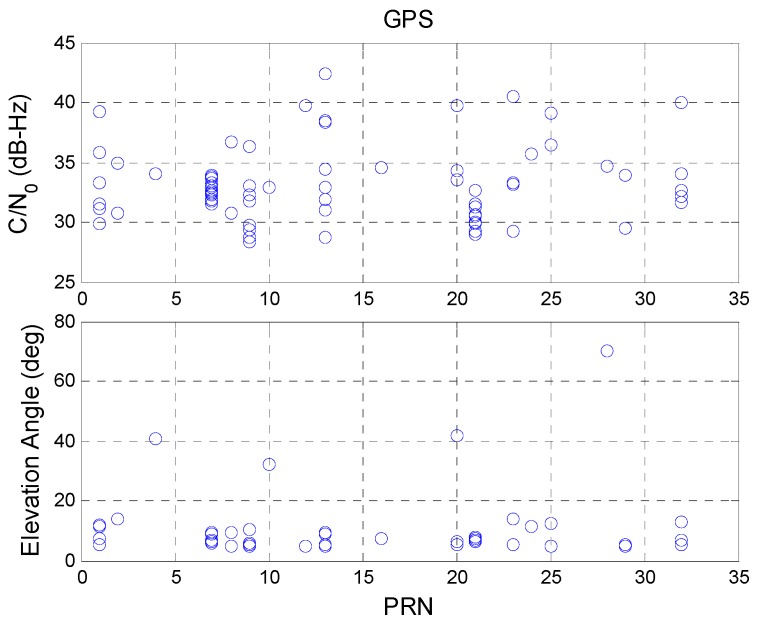
GPS cycle slips with respect to C/N_0_ and satellite elevation angle.

SQA3 is used to determine the time of cycle slips, which are then compared with the C/N_0_ values and elevation angles at a given moment. The above results (SQA1 and SQA2) are used to identify the reason for the signal discontinuity. The upper plot of Figure 11 shows the C/N_0_ value for each satellite during a cycle slip and the lower plot shows the satellite elevation angle at that moment. A comparison shows that most GPS signal cycle slips occurred when the satellite elevation angle was low, confirming the results of SQA2. If data for satellite elevation angles of below 20° are removed, the cycle slip interruption time for a whole day is only four seconds. These four cycle slips occurred when the satellite C/N_0_ value was irregularly low. Compared to other cycle slips results in this analysis, the four irregular satellites had a higher elevation angle. However, the signals from these satellites do not have the corresponding signal strength (all C/N_0_ values were below 40 dB-Hz). One possible reason is that the GNSS receiver might have been influenced by an interference signal at that moment. When signal interference occurs, the C/N_0_ value decreases due to the corresponding increase in signal noise. When there is no interference, cycle slips occur when the GPS satellites have a low elevation angle. As a result, the satellite elevation angle can be used as an indicator for GPS satellites to avoid cycle slips (in an open sky area).

**Figure 12:**
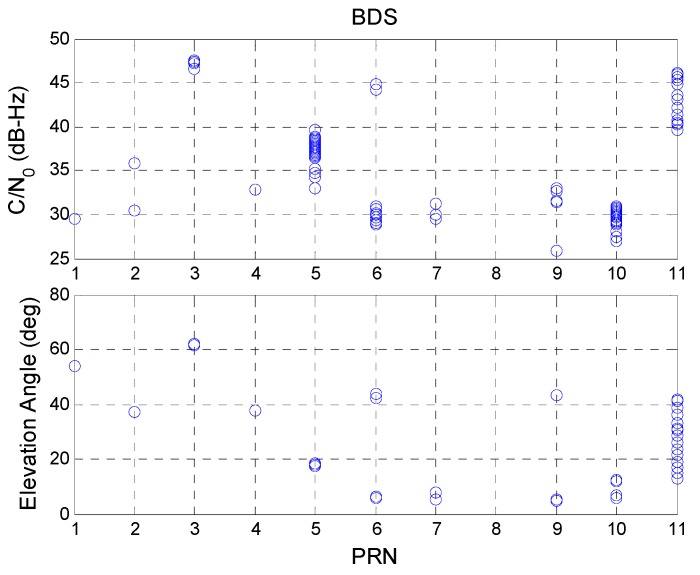
BDS cycle slip with respect to C/N_0_ and satellite elevation angle.

Figure 12 shows the SQA3 results for the BDS satellites. The BDS GEO PRN 5 satellite has the same result as those of the GPS satellites. For the PRN 5 satellite, the elevation angle is below 20° and most C/N_0_ values are below 40 dB-Hz. This explains why the BDS PRN 5 has lower continuity. However, PRN 3 had a high elevation angle and a high C/N_0_ value when a cycle slip occurred. This also occurred for BDS IGSO and MEO satellites. The high C/N_0_ value proves that there is no interference signal. This result shows that the BDS signal loses lock when the signal strength is good. This problem might be caused by the BDS satellite. Table 8 summarizes the final results for SQA3.

### 3.4. SQA Conclusions

Most of the time, a BDS user can receive a stable and continuous signal. All BDS C/N_0_ values are lower than those for GPS due to the transmission distances being longer for the former. However, even though the C/N_0_ values are lower for each elevation angle, the BDS GEO and IGSO signal strengths are more stable than those of GPS satellites. A BDS user can use the results of SQA1 to remove irregular satellites. SQA2 and SQA3 show that by removing the satellite with the highest chance of a cycle slip, BDS can provide a good-continuity signal. If a BDS user follows the proposed SQA processes to remove irregular satellites, the probability of incorrect range measurements generated by the receiver will decrease and the user position solution availability will increase.

## 4. Measurement Quality Analysis

When a signal is received and processed by the receiver, code-phase and carrier-phase measurements are generated. For precise positioning applications, algorithms such as the real-time kinematics (RTK) algorithm use carrier-phase measurements to calculate positioning results [25,28]. If the baseline length between two receivers is less than 10 km and the receivers are located at similar altitudes, the double difference algorithm can remove most of error in the measurements except receiver noise and multi-path error [29]. For the RTK algorithm, the stability of the measurement is most important. As a result, the only thing that needs to be analyzed is whether the stabilities of different GNSS measurements are on the same level. In order to achieve centimeter-level positioning, resolving integer ambiguity is the most important issue. A stable integer ambiguity solution requires a stable carrier measurement [30]. Therefore, analyzing the stability of the measurement is very important for precise positioning applications. For the general user, the code-phase measurement is accurate enough. However, the error sources in the ranging measurement, including satellite clock and orbit errors, ionospheric error, tropospheric error, multipath error, receiver clock bias, and receiver thermal noise, influence positioning performance [17]. By using the correction model, a GPS and BDS user can acquire more accurate positioning results. Unfortunately, the general models cannot remove all the errors in the ranging measurement [31]. Because the location of the reference station used in this research is known, an accurate range between the satellite and the receiver can be calculated. This research shows the ranging residual difference for GPS and BDS after using all models to remove the error sources. MQA is thus divided into four parts:

1. MQA1: Measurement stability analysis
2. MQA2: Double difference zero-baseline analysis
3. MQA3: Measurement error source analysis
4. MQA4: Measurement pseudorange residual analysis

The data used in this section are the same data used in Section 2, which were recorded on the 22nd of July 2014 for 24 h. The raw measurements are separated into code-phase pseudorange and carrier-phase observables. These two measurements were directly output by the receiver without any smoothing filter or data transformation. The data were recorded at a rate of 1 Hz. In this section, the statistical results were estimated using all the data. The total numbers of data points for GPS and BDS were 870,874 and 899,137, respectively.

### 4.1. MQA1: Measurement Stability Analysis

**Figure 13:**
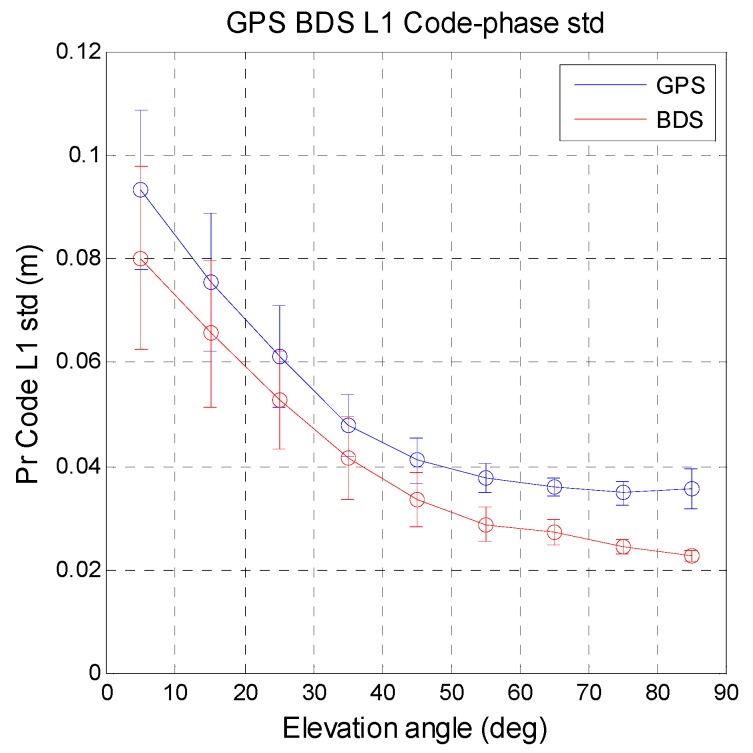
Relationship between code-phase pseudorange standard deviation and satellite elevation angle.

**Figure 14:**
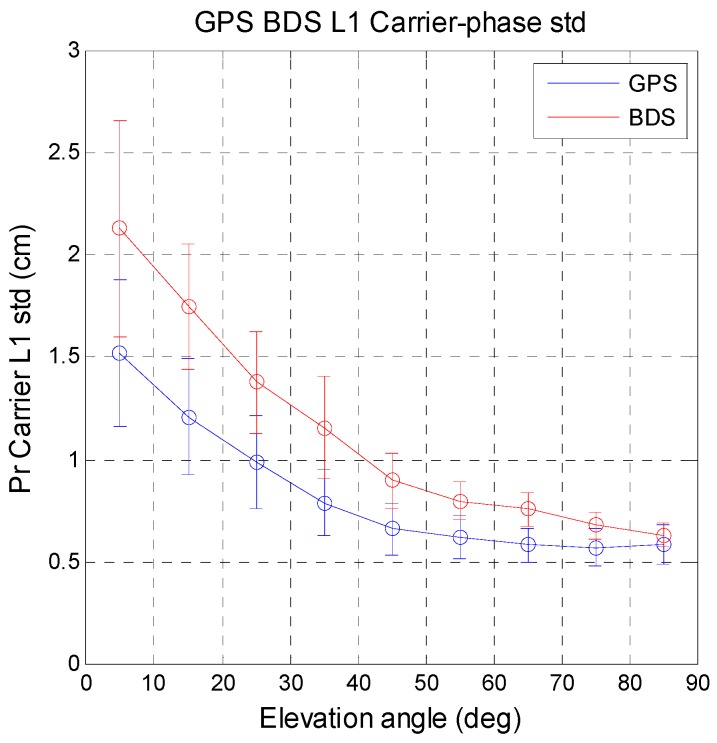
Relationship between carrier-phase pseudorange standard deviation and satellite elevation angle.

The measurement noise (*i.e.*, pseudorange standard deviation) is output from the receiver tracking loop. The details and the definition of the pseudorange standard deviation can be found at [32,33]. This value represents the measurement noise for the current data. The following analysis compares the constellation satellite measurement noise for a given receiver. Figure 13 shows the relation between code-phase pseudorange standard deviation and satellite elevation angle for 10° increments. The mean value and standard deviation of the pseudorange noise were calculated for the two systems. The circles in Figure 13 are the mean values and the vertical bars are the standard deviation values. The same analysis for the carrier-phase pseudorange noise is shown in Figure 14. Compared with GPS, BDS has less noise for all satellite elevation angles for the code-phase measurements. BDS provides more stable measurements for the user. The carrier-phase pseudorange noise for BDS is slightly higher than that for GPS. The differences between the two systems are less than 1 cm for middle to high elevation angles (50°–80°). The difference for the remaining angles is around 1 cm. Carrier-phase measurement stability is important for precise positioning. These results indicate that the code and carrier measurements of GPS and BDS have almost the same measurement noise. The statistical results for MQA1 are summarized in Table 9.

### 4.2. MQA2: Double Difference Zero-Baseline Analysis

Double difference zero-baseline analysis can be used to verify the precision of receiver measurements [29]. In this analysis, two GPS receivers were connected to the same antenna using a splitter (GPS Networking NALDCBS1X8 antenna splitter). When two receivers share an antenna, the error sources from the satellite signal (e.g., satellite clock, orbit, tropospheric, and ionospheric errors) are dependent. Because the signal paths are the same, the multipath errors will be the same for the two receivers. With one antenna used, the baseline is defined as zero. As a result, any nonzero result is generated by receiver noise. The quality of the zero-baseline result indicates the observation capability, measurement accuracy, and stability of the receiver.

**Figure 15:**
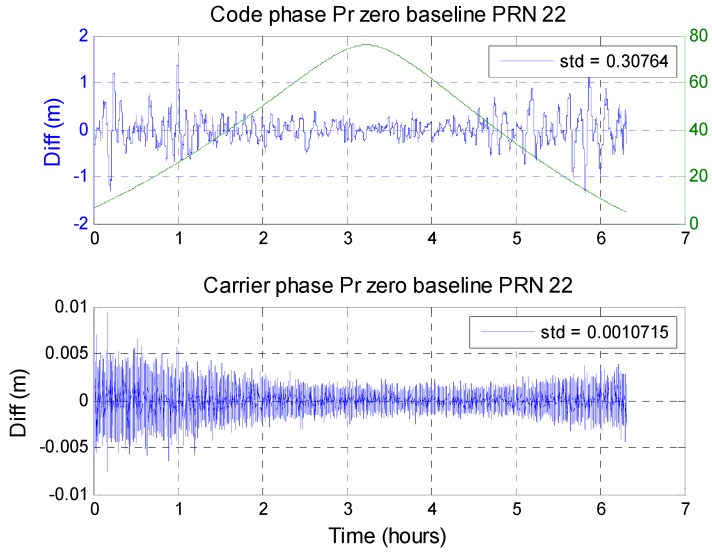
GPS PRN 22 zero-baseline code and carrier pseudorange difference.

**Figure 16:**
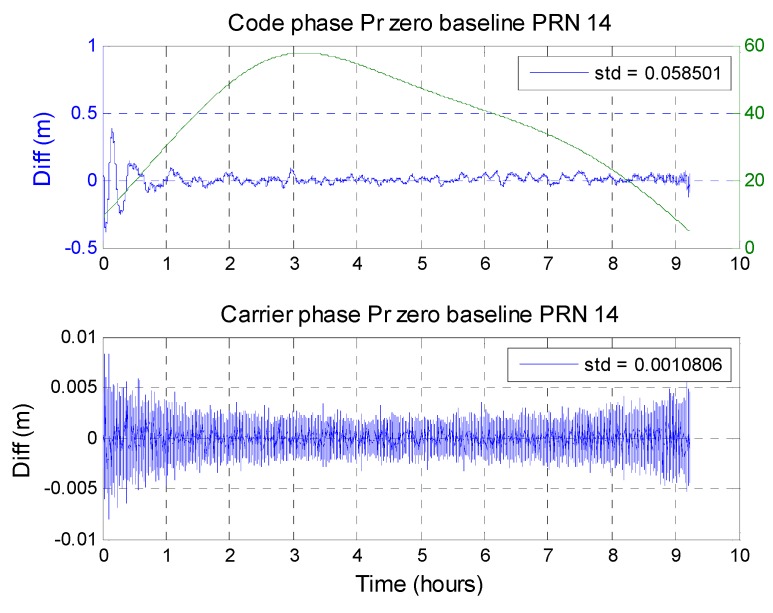
BDS PRN 14 zero-baseline code and carrier pseudorange difference.

To calculate the short baseline double difference values, the satellite with the highest elevation was selected as the main satellite. The double difference results for the remaining in-view satellites were calculated with respect to the main satellite. Figure 15 and Figure 16 show satellites with the most complete trends (from low elevation angle to high elevation angle and then to low elevation angle again) to show the zero-baseline results for GPS and BDS. Figure 15 shows the GPS PRN 22 zero-baseline result. The upper plot shows the zero-baseline result obtained using the code-phase pseudorange measurement from the two receivers. The two measurements from the same satellite go through a double difference calculation. The result is shown as the blue line. The green line is the elevation angle for this satellite. The lower plot shows the carrier-phase pseudorange measurement results. Both the code and carrier have higher noise when the satellite elevation angle is lower. However, the noise grades are not the same. For BDS, Figure 16 shows the BDS PRN 14 zero-baseline results. For a GPS satellite, the standard deviation of code-phase MQA2 results for all satellites is 29.46 cm and the carrier phase is 0.10 cm. The results are summarized in Table 10. The carrier-phase double difference zero-baseline results for the two systems are almost the same. However, in the code-phase pseudorange, the receiver generates more similar measurements for BDS.

### 4.3. MQA3: Error Source Analysis

**Figure 17:**
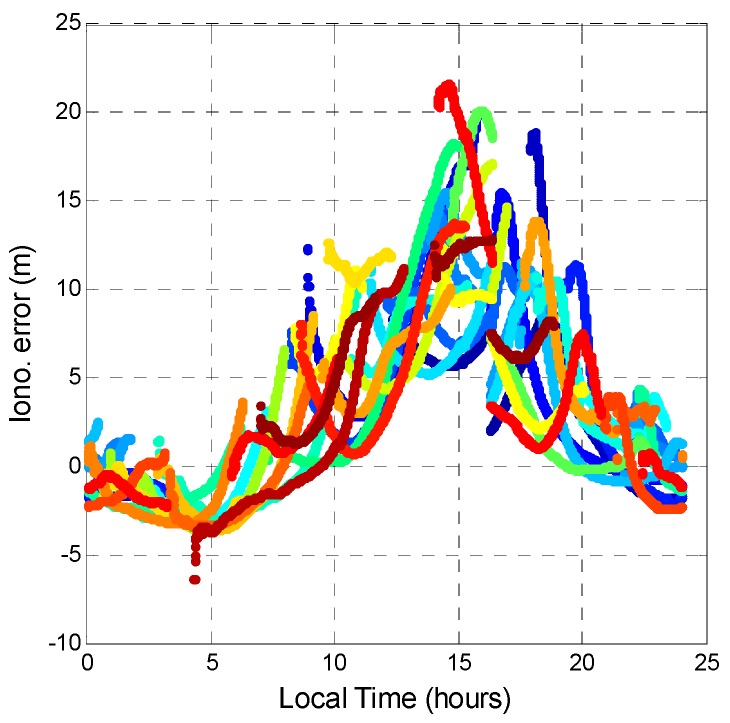
GPS ionospheric error during a day.

**Figure 18:**
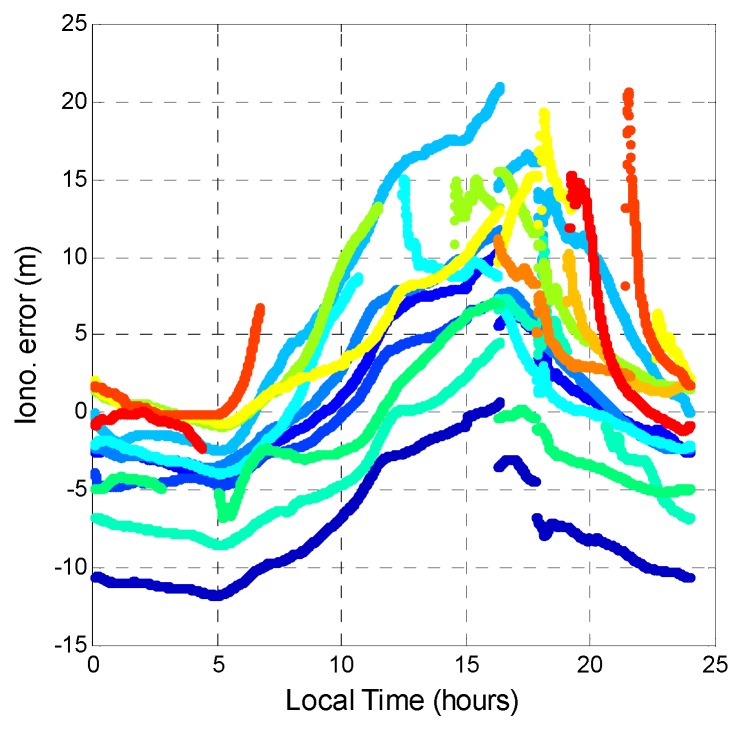
BDS ionospheric error during a day.

This section estimates the tropospheric delay, ionospheric delay, and receiver clock bias. First, the ionospheric error is generated by the GPS signal being refracted by free electrons in the ionosphere. The ionospheric delay was calculated from dual-frequency pseudorange measurements. This research utilizes the Hatch filter with a slightly modified ionospheric delay algorithm [31]. This ionospheric Hatch filter is used to estimate the smoothed ionospheric delay using the dual-frequency code and carrier measurements. In order to analyze the relationship between the error source and the local effect, the time axis was changed into the local time to analyze the ionospheric error. Figure 17 and Figure 18 show the ionospheric delay values for all GPS and BDS satellites, respectively. The colors represent the ionospheric delay value for each satellite. In Figure 17, the GPS ionospheric error varies with time. The maximum ionospheric delay occurs at 14:00 local time, and the minimum occurs at 04:00 local time. This result shows that ionospheric delay is related to solar activity. Figure 18 shows that the ionospheric error for BDS has the same tendency as that for GPS. However, at night (local time), the ionospheric delays of the GPS satellites converge to almost the same value no matter the satellite elevation angle. The BDS satellites have a larger variation range of the ionospheric delay during the night. The two systems have almost the same variation in ionospheric error. The maximum and minimum values during a day for GPS and BDS are similar. However, during night time, BDS has a dispersed ionospheric delay value. The distribution of BDS ionospheric error is larger than that of GPS for the whole day. It should be noted that the satellite differential code biases are not considered in the ionospheric delay computation shown in this paper, and the differences of the ionosphere delays in Figure 17 and Figure 18 could be due to satellite differential code biases.

**Figure 19:**
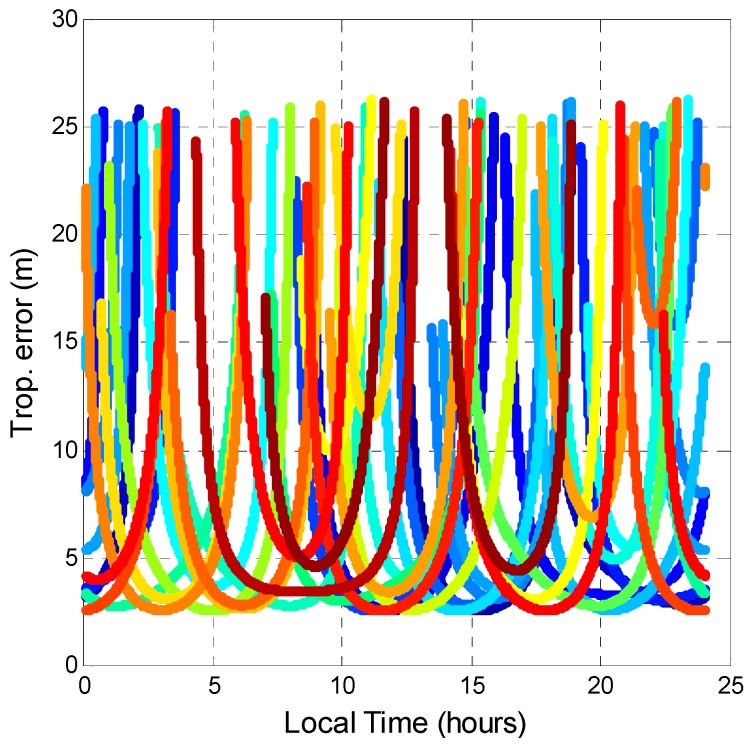
GPS tropospheric error during a day.

**Figure 20:**
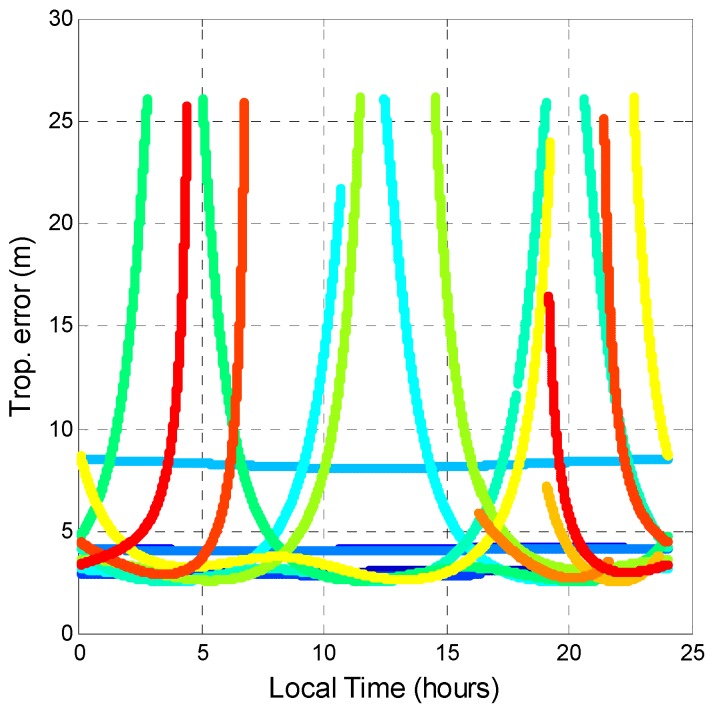
BDS tropospheric error during a day.

The tropospheric error was also analyzed. Tropospheric error is caused by the GPS signal passing through the troposphere. Variations in temperature, pressure, and humidity all contribute to variation in the speed of light of radio waves. In comparison to the vacuum speed of light, the delay value is the tropospheric error. Although some new mapping functions are available for estimating tropospheric delay [34,35], this research selected the Saastamoinen model [36] and the Black and Eisner troposphere formula [37] as the tropospheric delay model and mapping function, respectively, because they are the most commonly used in GNSS augmentation systems [31]. The input parameters for the Saastamoinen model was obtained from [38]. The tropospheric delays of all satellite pseudoranges were calculated. As shown in Figure 19 and Figure 20, the tropospheric delay value is inversely proportional to the satellite elevation angle. The maximum and minimum tropospheric delay values of GPS and BDS are almost the same. The horizontal line in Figure 20 represents the BDS GEO tropospheric delay. The tropospheric delay changed only slightly because GEO satellite elevation angles do not change much. As a result, by comparing different GNSS, the tropospheric corrections provided by the Saastamoinen model are almost the same.

**Figure 21:**
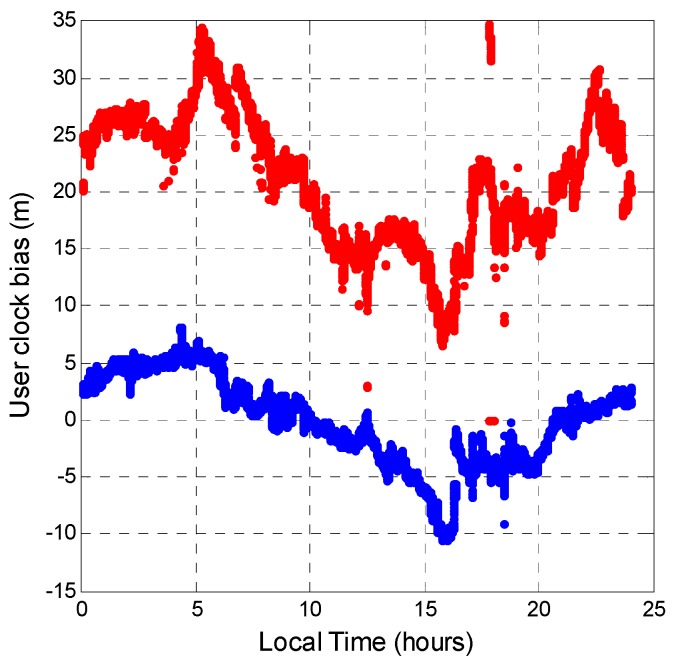
User clock bias (**Blue**: GPS clock bias; **Red**: BDS clock bias).

The user clock bias was analyzed next. Even if a user uses the same receiver to receive both GPS and BDS signals at the same time, the receiver clock offset is different for the two systems. Because the measurement generation of GPS and BDS is different, the user clock bias needs to be estimated separately. By using the modeled ionosphere and troposphere delays, the ionosphere- and troposphere-free pseudorange measurements are used to estimate the user clock bias. Figure 21 shows the user clock biases calculated for the two systems (blue points for GPS and red points BDS). The variation trends of these two clock biases are similar. The mean value of the clock bias difference between BDS and GPS is 21.070 m. The constant shift between these two systems is due to the measurement being generated using different standards. The BDS user clock bias has a larger standard deviation. With the ionospheric and tropospheric delays removed, the remaining error sources in the pseudorange measurement are satellite clock error, satellite orbit error, multipath error, and receiver thermal noise. Since this experiment used one receiver to receive both signals at the same time, the receiver thermal noise should be the same. Because the surrounding environment had no permanent obstructions (buildings) above the antenna site, multipath error was not considered. The BDS user clock bias is thus noisier due to satellite clock and orbit errors.

The main objective of this section is to present the estimation method for each error source. After that, remove these errors and generate a clean pseudorange residual to analyze the performance of the correction estimation in the next section.

### 4.4. MQA4: Pseudorange Residual Analysis

**Figure 22:**
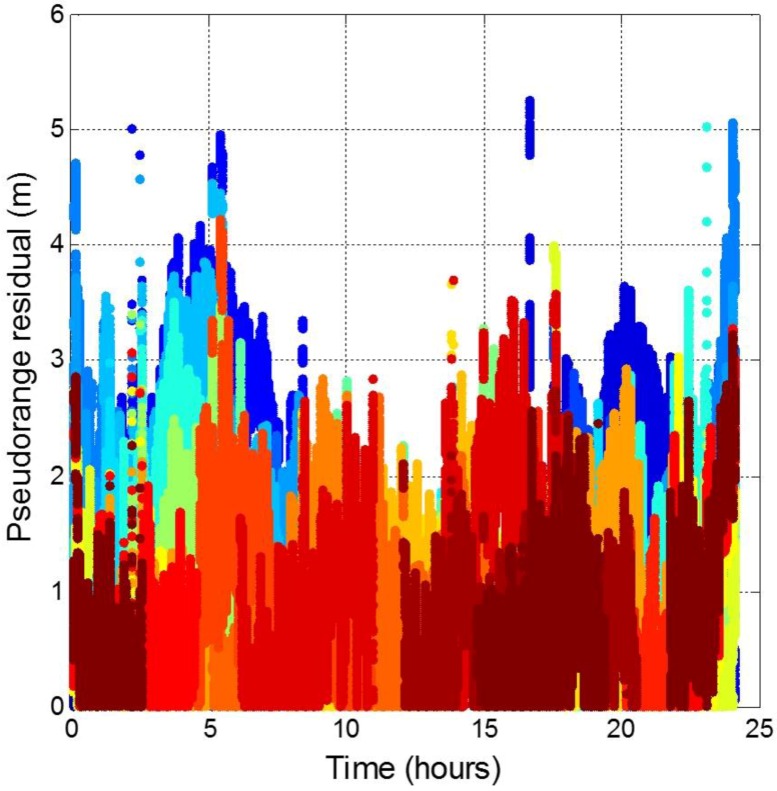
Pseudorange residuals for all GPS satellites.

The ephemeris data provide accurate satellite position estimation and satellite clock correction. In order to further analyze the satellite orbit and clock errors in the pseudorange, the pseudorange residual was used. First, the range between the known position and the satellite position calculated from the ephemeris was obtained. The definition of the pseudorange residual is the pseudorange that removed all the errors we calculate before (ionospheric, tropospheric, and user clock bias) and minus the range between the known user position and the estimated satellite position. The errors in the pseudorange residuals are the satellite orbit, satellite clock errors, multipath errors, and measurement noise. Figure 22 shows all GPS satellite pseudorange residuals (different colors stand for different satellites). The variation trend for each satellite is removing due to the error source which change with time has been removed. 95% of the GPS satellite orbit and clock errors is less than 2.53 m and the maximum error value is around 5 m. The statistical results are shown in Table 11.

**Figure 23:**
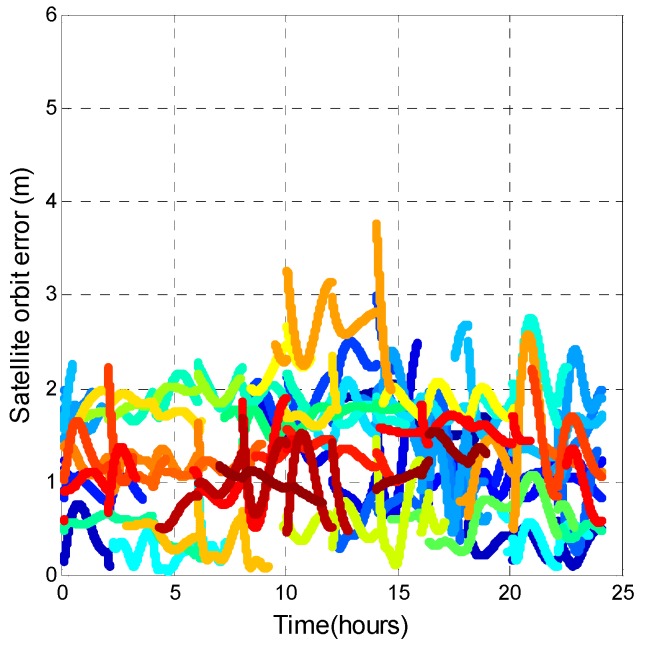
GPS satellite orbit error (compared with precise orbit correction).

The GPS precise ephemeris from the International GNSS Service (IGS) was applied to confirm our analysis results. However, no open source data supports precise ephemeris for BDS. Thus, only GPS satellite performance is analyzed in this section. Different from broadcast navigation data, the precise orbits are 3D satellite positions at the ECEF coordinate. The precise satellite position was recorded at intervals of 15 min. Thus, the user needs to interpolate the orbits between each precise satellite position. The interpolation method is from [39]. Figure 23 shows the corresponding satellite orbit error for Figure 22. The main error in the pseudorange residual is the satellite orbit error.

**Figure 24:**
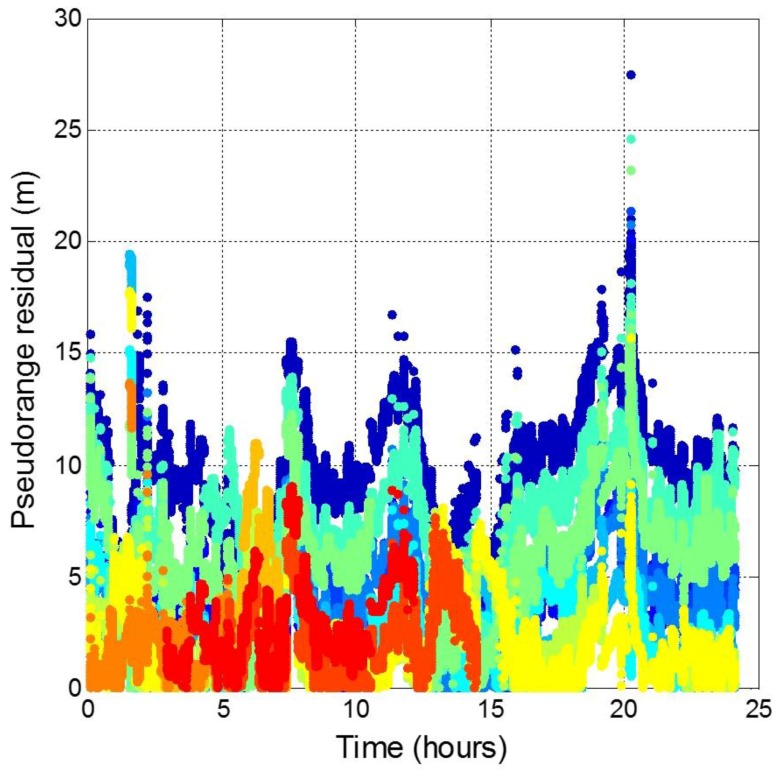
Pseudorange residuals for all BDS satellites.

**Figure 25:**
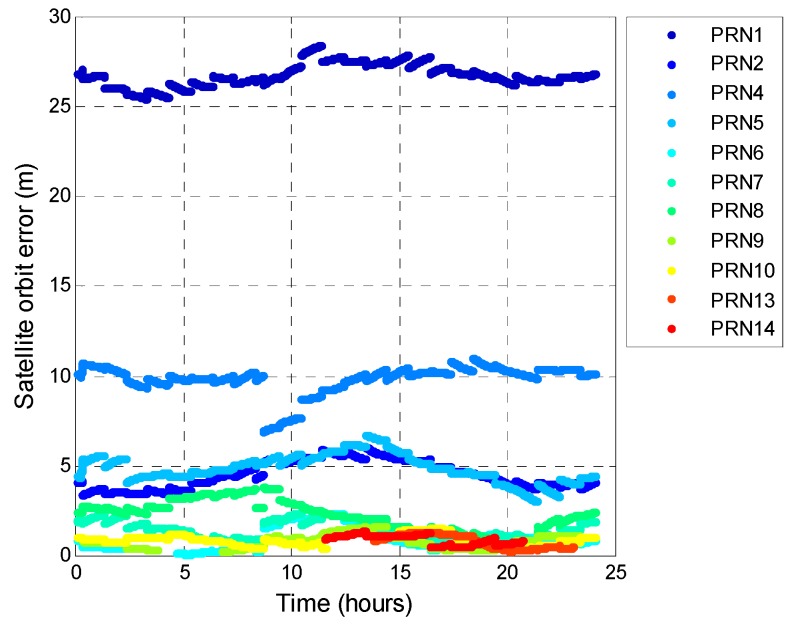
BDS satellite orbit error (compared with precise orbit correction).

The BDS satellite pseudorange residuals are plotted in Figure 24. By verifying with the precise orbit information from [40], the satellite orbit error is shown in Figure 25. The BDS PRN3, PRN11, PRN12 is lack of precise orbit information. The results show that 95% of the BDS satellite errors is below about 10 m. The maximum value is over 25 m. The BDS ephemeris error is thus larger than that for GPS. This result indicates that the satellite control and prediction of BDS faces some issues. For real-time positioning, the pseudorange residual is proportional to the positioning error. As a result, the satellite orbit and clock errors are the main reason that BDS could not provide the service as good as that of GPS.

### 4.5. MQA Conclusions

The measurement stability and error were analyzed for both systems. The measurement stability for BDS code-phase measurements is better than that for GPS and the carrier-phase measurements have almost the same performance. However, the BDS satellite orbit and clock errors are larger in the BDS pseudorange. This result confirms the DQA results. Referring to DQA1 and 2, the error from satellite clock is larger than the satellite orbit error for the general case. Applying extra satellite clock correction estimation to have a more accurate pseudorange increases the compatibility and interoperability between GPS and BDS.

## 5. Conclusions

This study analyzed actual signal in space data, signals, and measurements for GPS and BDS. In order to remove undesired data and provide more accurate positioning results for both systems, each QA established the different statistical result. The satellite observations are independent for each analysis, with different means and standard deviations. The threshold is derived from the Gaussian distribution, which approximates the distribution of satellite observations. In order to establish a suitable threshold for each analysis, the threshold is defined as *μ + kσ*, where *μ* and *σ* are the mean and standard deviation of the established statistical results, respectively, and *k* is a value that can be defined by the user for different positioning applications. By using satellites that pass each QA’s threshold, unusual measurement errors can be avoided.

Three quality analyses were used to determine the performance difference between GPS and BDS. More than one month of GNSS data were used for verification. It was found that the performance of BDS is almost the same as that of GPS most of the time. BDS provides more stable code-phase measurements and signal strength than those of GPS. However, sometimes BDS broadcasts irregular ephemeris and almanac data information. The satellite orbit and clock errors cannot be determined using a conventional GNSS receiver. The proposed quality analyses can be used to detect these anomaly errors. Finally, the suggestions given in this study will facilitate the integration of GPS and BDS.

## Figures and Tables

**Table 1:** System comparison between GPS and BDS [2,11].

	GPS	BDS		
**Orbit**	MEO	GEO	IGSO	MEO
**Orbit Radius (km)**	20,200	35,786	35,786	21,528
**Inclination**	55°	<2°	55°	55°
**Sat. Number (Until 2014)**	31	5	5	4
**Planned Sat. Number**	24	5	3	27
**PRN Number**	1~32	1~5	6~10	11~14
***μ* (m^3^/s^2^)·10^14^**	3.986005	3.986004418		
***Ω_e_* (rad/s)·10^−5^**	7.2921151467	7.2921150		
**Coordinates**	WGS84 *	CGCS2000 *		
**Time**	GPST **	BDT **		
**Time Start**	6 January 1980	1 January 2006		
**1st Carrier**	1575.42 MHz	1561.098 MHz		
**2nd Carrier**	1227.6 MHz	1207.14 MHz		

* WGS84: World Geodetic System 1984; CGCS2000: China Geodetic Coordinate System 2000; ** GPST: Global Positioning System Time; BDT: BeiDou Navigation Satellite System Time.

**Table 2:** DQA1 maximum (Max), mean value (Mean), and standard deviation (Std) results of satellite position difference between using original and new ephemeris (unit: m).

	GPS	BDS		
**Orbit**	MEO	GEO	IGSO	MEO
**Max**	2.01	3.41	27.78	54.28
**Mean**	0.50	0.39	0.41	3.28
**Std**	0.30	0.26	0.77	5.76

**Table 3:** DQA2 statistical results of satellite clock bias in pseudorange domain (unit: m).

	GPS	BDS		
**Orbit**	MEO	GEO	IGSO	MEO
**Max**	0.35	1.70	1.88	2.85
**Mean**	−0.001	0.005	−0.01	0.10
**Std**	0.12	0.20	0.32	0.73

**Table 4:** DQA4 statistical result of satellite position difference between using new almanac and new ephemeris (unit: m).

	GPS	BDS		
**Orbit**	MEO	GEO	IGSO	MEO
**Max**	4982.2	4028.6	4448.9	3463.3
**Mean**	1537.8	1960.9	1937	1273.9
**Std**	716.4	834.9	831.7	575.3

**Table 5:** DQA5 satellite velocity (vel.), position (pos.), and elevation angle (ele.) in fifth week.

	GPS	BDS		
**Orbit**	MEO	GEO	IGSO	MEO
**Max Vel. Error (m/s)**	18.71	2.75	147.73	10.50
**Max Pos. Error (m)**	161,347	328,923	2,188,705	81,091
**Max Ele. Error (°)**	0.427	0.292	2.633	0.189

**Table 6:** SQA1 statistical results.

**Ele.<**	10	20	30	40	50	60	70	80	90
**GPS**									
**Mean**	41.3	43.2	45.3	47.5	48.7	49.4	49.9	50.1	49.9
**Std**	2.0	1.6	1.2	1.0	0.8	0.6	0.4	0.6	0.8
**BDS GEO**									
**Mean**	NaN	38.7	NaN	42.9	NaN	47.0	47.0	NaN	NaN
**Std**	NaN	0.8	NaN	1.5	NaN	0.3	0.2	NaN	NaN
**BDS IGSO**									
**Mean**	36.6	38.2	40.2	42.8	44.2	45.4	46.6	47.3	47.6
**Std**	2.1	1.3	1.1	1.1	0.7	0.9	0.6	0.5	0.2
**BDS MEO**									
**Mean**	40.0	41.4	43.2	45.7	47.1	47.9	48.3	48.5	48.5
**Std**	1.4	1.6	1.3	1.2	1.0	0.8	0.6	0.8	0.3

**Table 7:** SQA2 statistical results.

	GPS	BDS			
**Orbit**	MEO	GEO 1–4	GEO 5	IGSO	MEO
**Mean Continuity (%)**	>99.99	>99.99	99.86	99.98	99.97
Mean CSN *	2.5	2.75	115	9.6	3.8

* CSN: Cycle slip number.

**Table 8:** SQA3 statistical results.

	GPS	BDS		
**Orbit**	MEO	GEO	IGSO	MEO
**Mean C/N_0_**	34.1	36.0	30.7	43.3
**Mean Ele. (°)**	15.4	41.7	9.4	27.7

**Table 9:** MQA1 statistical results.

**Ele.<**	10	20	30	40	50	60	70	80	90
**GPS L1 Code**									
**Mean**	9.35	7.58	6.12	4.79	4.12	3.78	3.59	3.48	3.57
**Std**	1.53	1.33	0.97	0.59	0.44	0.27	0.18	0.24	0.38
**GPS L1 Carrier**									
**Mean**	1.52	1.21	0.99	0.79	0.66	0.62	0.58	0.57	0.58
**Std**	0.36	0.28	0.23	0.16	0.12	0.11	0.08	0.09	0.10
**BDS L1 Code**									
**Mean**	8.03	6.57	5.27	4.16	3.36	2.87	2.72	2.43	2.26
**Std**	1.78	1.42	0.95	0.81	0.52	0.33	0.23	0.15	0.10
**BDS L1 Carrier**									
**Mean**	2.13	1.75	1.38	1.16	0.90	0.80	0.76	0.68	0.63
**Std**	0.53	0.30	0.25	0.25	0.14	0.09	0.09	0.07	0.05

**Table 10:** MQA2 statistical result (unit: m).

	GPS	BDS		
**Orbit**	MEO	GEO	IGSO	MEO
**L1 Code**	29.46	5.54	4.52	4.86
**L1 Carrier**	0.10	0.13	0.13	0.10

**Table 11:** MQA4 statistical results (unit: m).

	GPS	BDS		
**Orbit**	MEO	GEO	IGSO	MEO
**Mean**	0.98	4.84	4.06	2.45
**Std**	0.78	3.53	3.11	2.04

## Abbreviations

The following abbreviations are used in this manuscript:

GNSS	Global Navigation Satellite System
BDS	BeiDou Navigation Satellite System
GPS	Global Positioning System
SIS	Signal in space
DQA	Data quality analysis
SQA	Signal quality analysis
MQA	Measurement quality analysis
GLONASS	Global Navigation Satellite System
QZSS	Quasi-Zenith Satellite System
MEO	Medium Earth orbit
GEO	Geostationary Earth orbit
IGSO	Inclined geosynchronous orbit
CGCS2000	Chinese Geodetic Coordinate System 2000
WGS84	World Geodetic System 1984
BDT	BeiDou Navigation Satellite System Time
UTC	Universal Time
GPST	GPS Time
C/N_0_	Carrier-to-noise density ratio
RTK	Real-time kinematics
